# Fatty Liver Disease Prediction Model Based on Big Data of Electronic Physical Examination Records

**DOI:** 10.3389/fpubh.2021.668351

**Published:** 2021-04-12

**Authors:** Mingqi Zhao, Changjun Song, Tao Luo, Tianyue Huang, Shiming Lin

**Affiliations:** ^1^School of Mathematical Sciences Xiamen University, Xiamen, China; ^2^Department of Computer Engineering, Changji University, Changji, China; ^3^School of Informatics Xiamen University (National Demonstrative Software School), Xiamen, China

**Keywords:** fatty liver disease, electronic medical records, genetic algorithm, machine learning, XGBoost, chi-square binning algorithm

## Abstract

Fatty liver disease (FLD) is a common liver disease, which poses a great threat to people's health, but there is still no optimal method that can be used on a large-scale screening. This research is based on machine learning algorithms, using electronic physical examination records in the health database as data support, to a predictive model for FLD. The model has shown good predictive ability on the test set, with its AUC reaching 0.89. Since there are a large number of electronic physical examination records in most of health database, this model might be used as a non-invasive diagnostic tool for FLD for large-scale screening.

## 1. Introduction

Fatty liver disease (FLD) is a lesion with excessive accumulation of fat in liver cells, which is divided into non-alcoholic fatty liver disease (NAFLD) and alcoholic fatty liver disease (AFLD) (1). In recent years, with the improvement of living standards, changes in lifestyle and diet, and the wide use of ultrasound and other imaging technology, the prevalence of FLD is growing rapidly (2). In fact, it has become the most common cause of chronic liver disease in developed and developing countries (3). According to research, about 25% of people worldwide and 21% of people in China catch NAFLD (4, 5).

At present, the pathogenesis of NAFLD is not completely clear, and there is no ideal and effective treatment drug, but it is reversible in the early stages. Research shows that effective lifestyle interventions such as energy restriction, dietary changes, and increased physical activity are particularly effective in the early stages of NAFLD (6). Therefore, early detection and treatment is the key. At present, the main clinical diagnostic methods are ultrasound, CT, and liver biopsy (7). For their invasiveness and complexity, they are not suitable for large-scale epidemiological screening (8–10).

Based on the above situation, many scientists try to use machine learning algorithm to build the prediction model of FLD. In recent years, several machine learning models based on medical data have been proposed (11–13). Italian scholar Giorgio Bedogni collected data by gender, age, alcohol intake, alanine aminotransferase, aspartate aminotransferase, body mass index (BMI), waist circumference, the sum of four skinfolds, etc., and established a prediction model for NAFLD (13). However, most of the models are carried out through questionnaire surveys and medical experiments and use some features that are not easy to obtain in large quantities. The limitation of data quantity and the complexity of features make these models difficult to generalize.

The purpose of this study is to establish an efficient and convenient FLD prediction model using machine learning algorithm which can help doctors to screen out the patients that need further liver examination and can be applied to large-scale epidemiologic screening. To facilitate the generalization of the model, the features we use will be as convenient as possible, and the amount of data we use will be as much as possible.

## 2. Materials and Methods

### 2.1. Dataset

The development of the medical system, the popularity of electronic physical examination records, and the establishment of health databases provide data support for large-scale epidemiological research. The data set used in this study is from the health database of a hospital in China, which contains the electronic physical examination records of 44,854 patients. And in this data set, no patient's privacy information is included, only routine physical examination data and age are included. To simplify and generalize the model, we only extracted 129 routine physical examination items of all patients, including blood routine, biochemistry, urine routine, etc.

In this study, patients diagnosed with FLD by ultrasound were marked as 1, and the remaining patients were marked as 0. The prevalence of FLD in the data set is 23%, which is close to the previous research (5).

### 2.2. Data Preprocessing

Firstly, for the accuracy of the model, we deleted individuals who had not undergone ultrasound examination because we did not know if they had FLD. Then, we delete the items with more than $\frac{2}{3}$ missing values that most people have not been examined. Finally, we randomly select 70% of the data set as the training set of the model and 30% as the test set.

**Figure 1:**
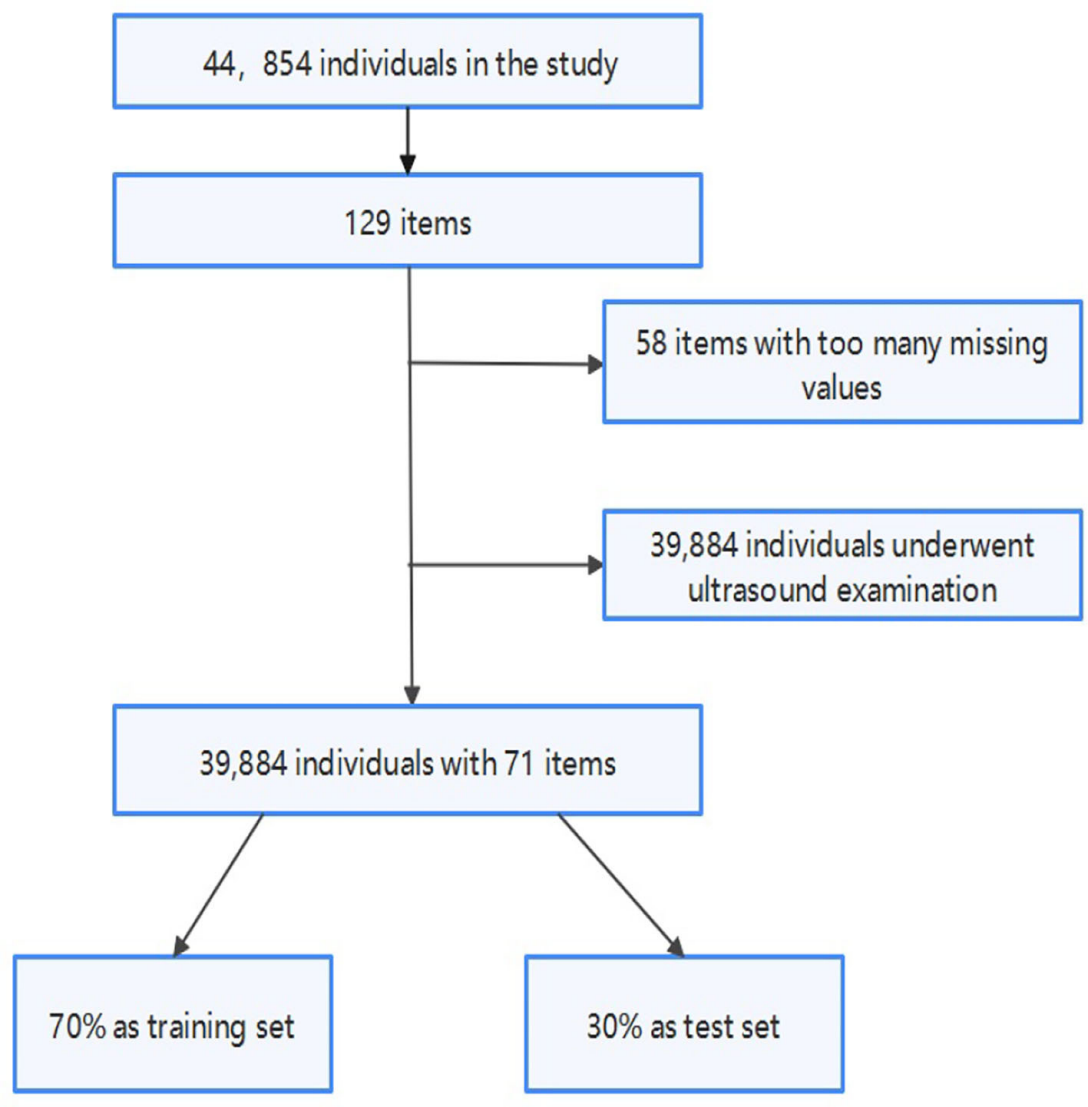
Data preprocessing flowchart.

**Figure 2:**
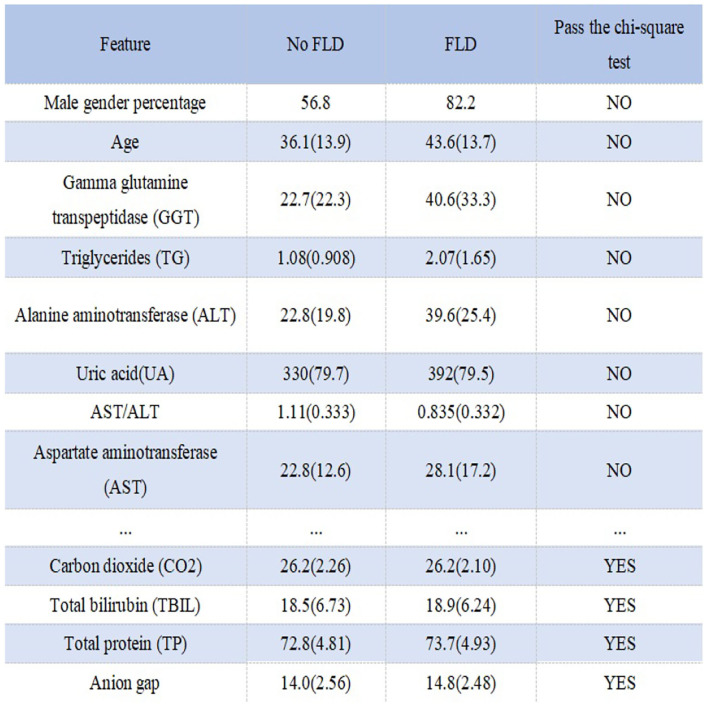
Statistical information and chi-square test results of different features in different groups.

Figure 1 shows the process of data preprocessing. Figure 2 shows the mean (standard deviation) of the different features of FLD patients and normal people and whether these features have passed the chi-square test with significance level of 0.05. It can be seen that there are significant differences in Male gender percentage, Uric acid (UA), Triglycerides (TG), Alanine aminotransferase (ALT), Aspartate aminotransferase (AST), Gamma glutamine transpeptidase (GGT), Age and AST/ALT between FLD patients and normal people, while Carbon dioxide (CO_2_), Total bilirubin (TBIL), Total protein (TP), and Anion gap do not.

### 2.3. Missing Value Processing

Compared with conducting medical experiments and questionnaire surveys, the advantage of using electronic physical examination records in the health database for modeling is that the amount of data is large and the model is easy to be generalized, but the disadvantage is that there are lots of missing values. Therefore, how to fill in missing values is critical to modeling. The usual practice is to fill in the mean or median for missing values. In fact, the distribution of medical indicators varies with gender and age, and the range is large. So it's a good choice to fill in the median according to age and gender.

For age grouping method, standard age grouping can be used, but the result is not ideal. So we use the chi-square binning algorithm to group age. Chi-square binning algorithm is a binning algorithm based on the chi-square test, which is specifically implemented by the independence test in the chi-square test. The theoretical basis for binning is: the lower the chi-square value between two bins, the more likely they are to have similar distributions (14). If two adjacent bins have very similar distributions, then the two bins should be merged, otherwise, they should be separated. Therefore, in each step of the algorithm, the two bins with the smallest chi-square value must be combined until the number of bins meets the stopping condition.

**Figure 3:**
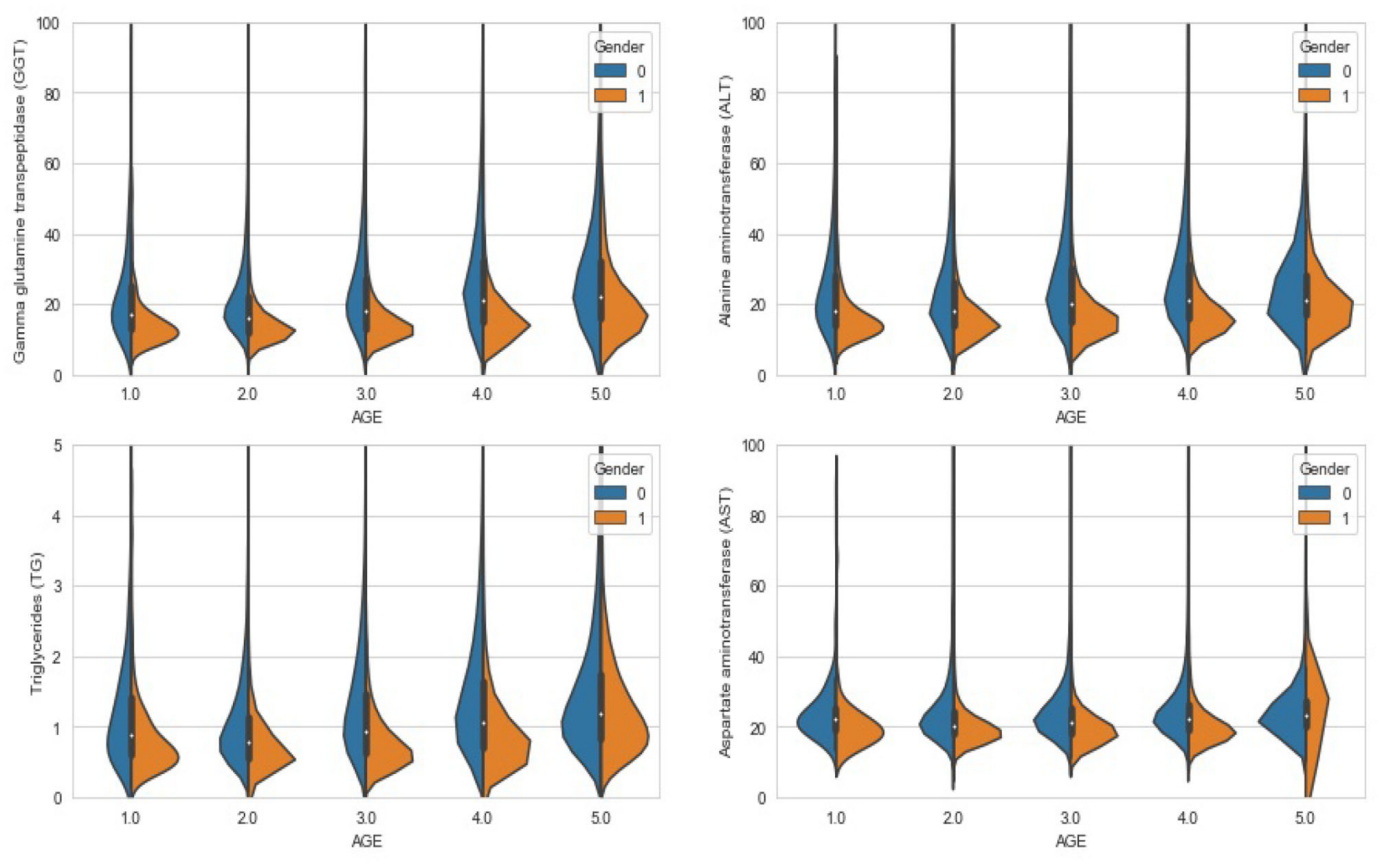
Violin chart: the distribution of different features under different age groups and genders.

In the present study, a bin refers to an age group and distribution refers to the prevalence of FLD. And we set the expected number of bins to 5, and the result after calculation on the training set is: [0, 17], (17, 29], (29, 35], (35, 47], (47, 197]. According to the results of age grouping, Figure 3 shows the distribution of several important features that need to be filled with missing values under different age and gender groups. It can be seen that the difference in distribution is obvious, so our strategy of filling in missing values is necessary and effective.

### 2.4. Feature Engineering

In machine learning modeling, the quality of features often determines the upper bound of model performance. Therefore, we need to do feature engineering on the existing routine features to maximize the usage of them. In clinical diagnosis, the combination of multiple characteristics often plays an important role in the judgment of diseases. For example, AST/ALT (Aspartate aminotransferase/Alanine aminotransferase) is of great significance in the diagnosis of liver diseases (1). So we want to generate new features through a combination of features.

In the present study, we use Spearman's correlation coefficient as a standard to measure the quality of features and use the genetic algorithm to find the optimal solution. Spearman's correlation coefficient, also known as rank correlation coefficient, can measure the rank correlation between two variables. If the machine learning model used is based on a decision tree, the Spearman correlation coefficient can measure the correlation between a feature and the target. The genetic algorithm is a method of searching for the optimal solution by simulating the natural evolution (15, 16). The algorithm transforms the problem-solving process into a process similar to the crossover and mutation of chromosomal genes in biological evolution. When solving more complex combinatorial optimization problems, Compared with some conventional optimization algorithms, it can usually obtain better optimization results faster (16).

**Figure 4:**
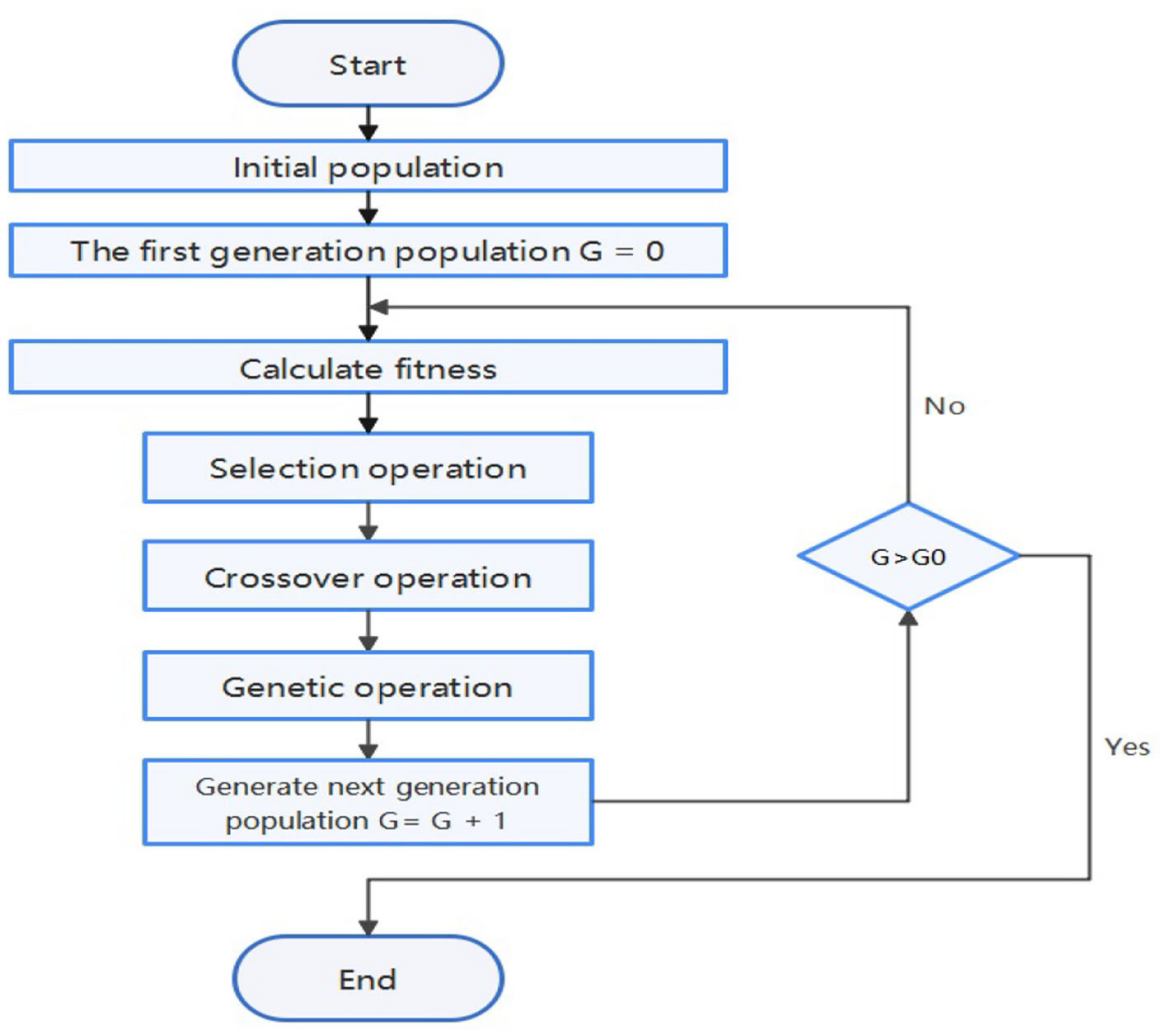
Genetic algorithm flowchart.

**Figure 5:**
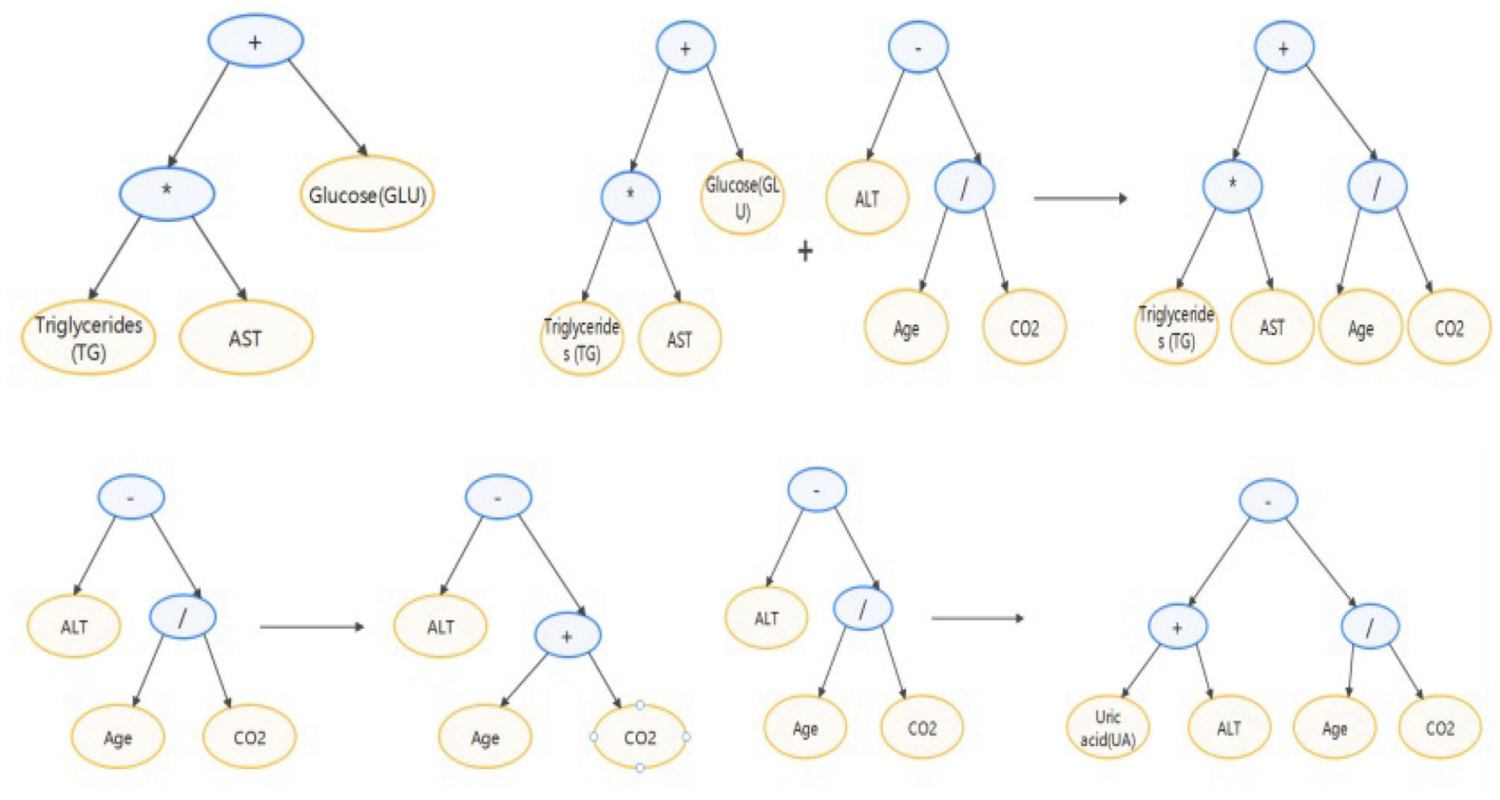
Demonstration of individual and individual variation.

Figure 4 shows the process of feature engineering using genetic algorithm. In the algorithm, an individual in the population is defined as a binary tree. Each leaf node of the binary tree is a certain feature in the data set, and each inner node of the binary tree is an operator in {+, -, *, /, log, sqrt}. Each individual represents an expression composed of features and operators. Fitness is the Spearman correlation coefficient between the new feature and the target. In each generation, individuals with high fitness will be retained, and individuals with low fitness will be eliminated. The upper left of Figure 5 shows an individual example, which represents TG*AST+*GLU*. The upper right and lower parts of Figure 5 respectively show crossover operations and mutation operations, both of which generate new individuals by changing subtrees in the way that simulates biological variation.

We set the number of individuals in each generation to 1,000 and set the maximum depth of the binary tree to three. Use the normalized features and iterating ten generations, the individuals with the first three fitness levels are added to the data set as new features. The result is:*GA*_*fea*1 = *TG*+*log*(*ALT*) *with fitness* 0.89, *GA*_*fea*2 = *TG* * *GGT with fitness* 0.87, and *GA*_*fea*3 = (*UA* + *AST*) * *log*(*ALT*) *with fitness* 0.79.

## 3. Experiments and results

XGBoost (eXtreme Gradient Boosting) is an engineering implementation of gradient boosting decision tree (GBDT) (17). Its core idea is to perform a second-order Taylor expansion of the loss function, and gradually train the decision tree based on the objective function, and greatly improve the training model speed (18, 19). XGboost has many advantages. For example, traditional GBDT only uses first-order derivative information in optimization, while XGboost performs a second-order Taylor expansion on the cost function to make the result more accurate. Xgboost adds a regular term to the cost function to control the complexity of the model, which reduces the variance of the model and makes the learned model simpler and prevents overfitting. XGboost supports parallel computing on feature granularity, which greatly reduces the amount of calculation and improves the training speed. In addition, XGBoost is a model based on the decision tree model, it is more explanatory than neural networks and other algorithms, which can enable us to better understand how a physical examination data plays a role in the model (20). Therefore, the present study uses the XGBoost model for modeling.

The error of a machine learning model includes two aspects: variance and bias (21). High bias models usually have relatively simple parameter settings and tend to underfit, that is, there is little difference in performance between the training set and test set, but both are relatively low. High variance models usually have complex parameter settings and tend to overfit. They perform well on the training set, but the performance on the test set drops seriously. The usual practice is to make a trade-off between variance and bias to get a reasonable model. AUC (Area Under Curve) is defined as the area under the ROC curve (Receiver Operating Characteristic curve), which is a commonly used indicator to measure the quality of a machine learning model (22). AUC has nothing to do with the ratio of positive and negative samples, it represents the model's ability to sort samples to a certain extent (23). In present study, we use AUC as the evaluation criterion of the XGBoost model. On the training set, Bayesian optimization of hyperparameters is performed using triple cross-validation, and then the obtained results are fine-tuned to prevent over-fitting and ensure the rationality of the parameters. The main results are as follows: *max*_*depth* : 3, *learning*_*rate* : 0.07, *n*_*estimators* : 150, *scale*_*pos*_*weight* : 2, *min*_*child*_*weight* : 6, *gamma* : 0.2, *reg*_*alpha* : 0.1.

**Figure 6:**
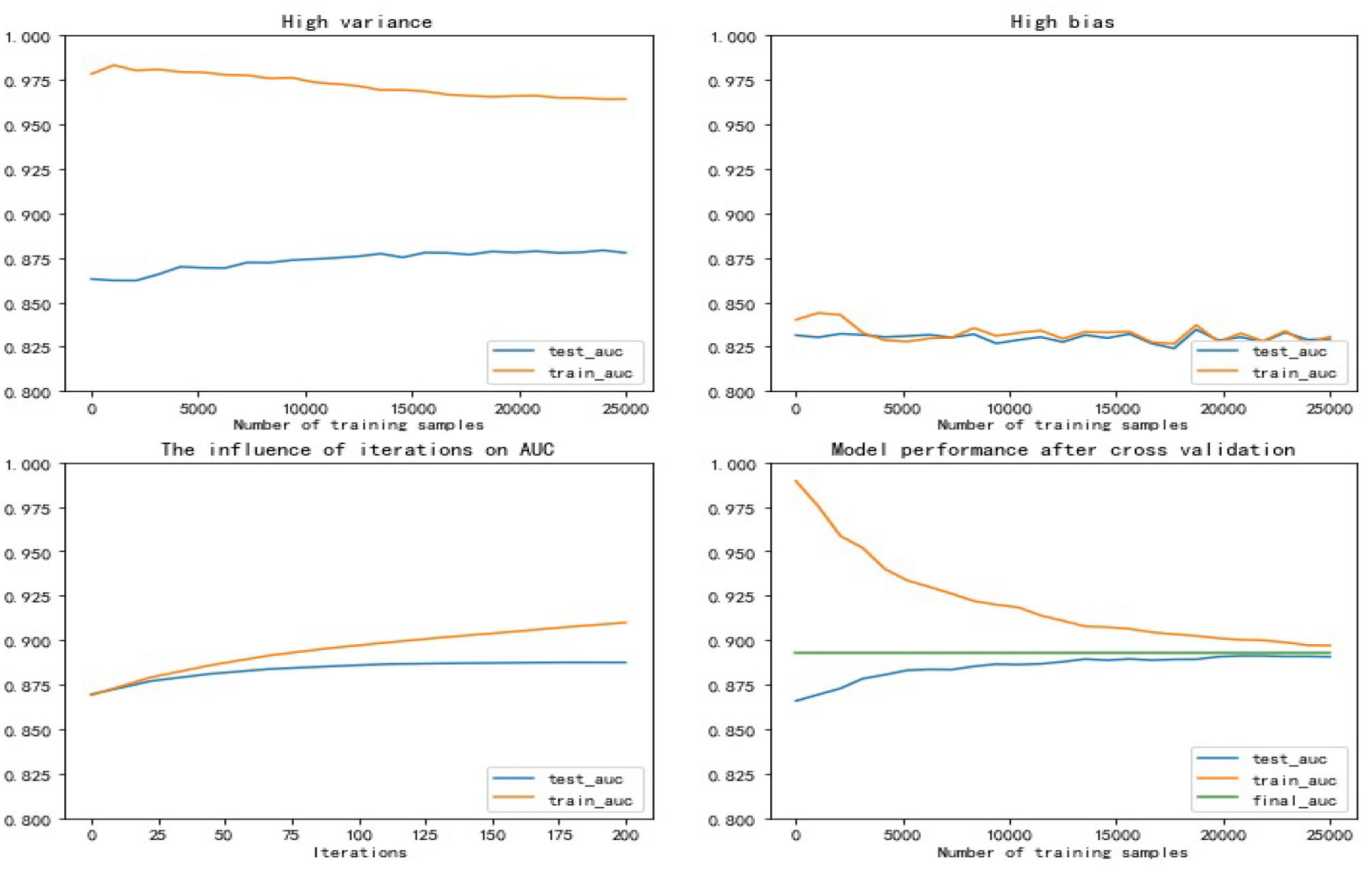
Trade-off between variance and bias.

The upper left and upper right of Figure 6 respectively show the performance of the high variance model and the high bias model. The lower left shows the effect of the hyperparameter *iterations* on the model performance. It can be seen that with the increase of *iterations*, the over fitting phenomenon of the model appears, and the variance of the model becomes larger. The lower right shows the performance of the model with the optimal hyperparameter combination set. It can be seen that the AUC of the model reached 0.89, which shows that the model has a strong predictive ability for FLD, and the performance of the model in the test set and training set is basically the same, without over fitting phenomenon.

## 4. Discussion

**Figure 7:**
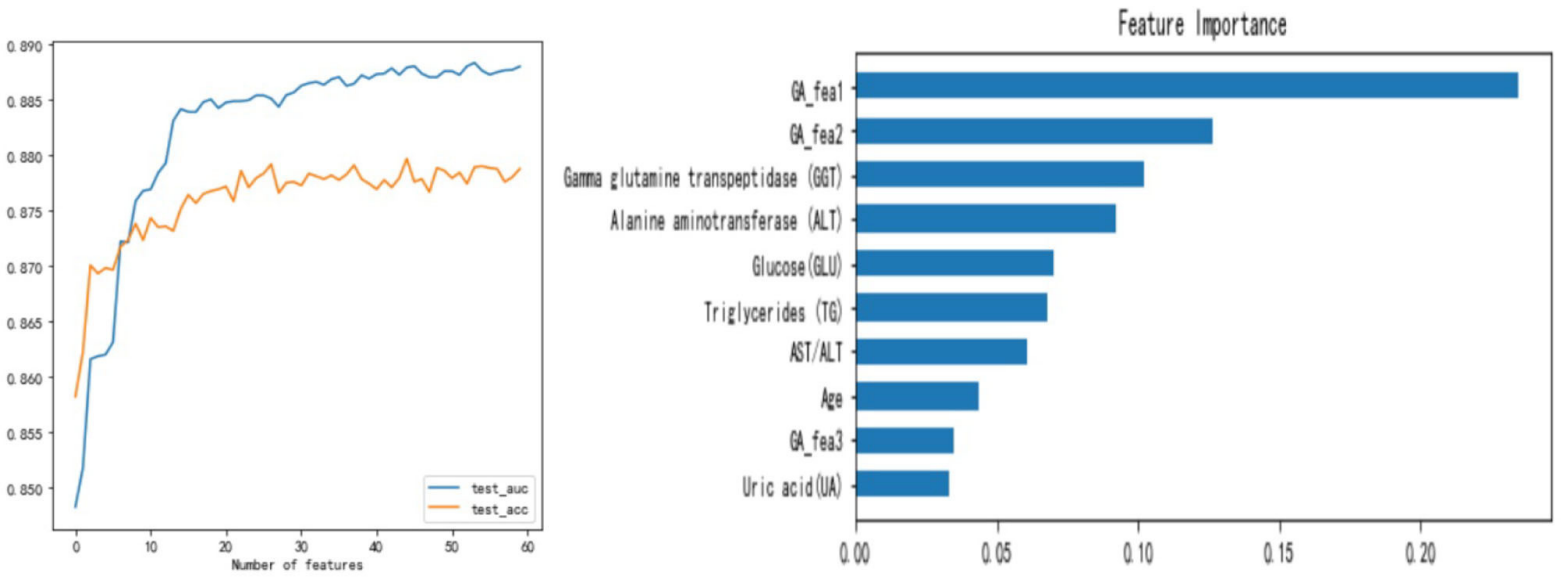
The influence of the number of features used on the model and feature importance.

Using the number of times the feature is used as the basis for splitting in the decision tree splitting as the importance of the feature, we can sort all the features by importance. Left of Figure 7 shows the model performance obtained by gradually adding the top 60 features of importance to the model. It can be seen that the top 10 features are the most important, and the features after the 20th place are dispensable. This shows that even if we only use the first ten features to train the model, its AUC can still reach the level of 0.87–0.88, but the model is greatly simplified at this time.

Right of Figure 7 shows the importance of the top 10 features. According to research, the degree of fat accumulation in the liver is directly proportional to body weight. The prevalence of obesity in NAFLD patients is estimated to be 51.34% (95% CI: 41.38–61.20) (1), so many FLD patients have a significant increase in TG. At the same time, and when liver disease occurs, ALT and GGT will increase significantly. Right of Figure 7 shows that TG, ALT, GGT, *GA*_*fea*1, and *GA*_*fea*2 play a vital role in the model, which is in line with the facts. Studies have also shown that the prevalence of diabetes in NAFLD patients is estimated to be 22.51% (95%CI: 17.92–27.89) (1), and with the increase of age, people's metabolism slows down and people are more likely to suffer from metabolic diseases. So the importance of GLU and Age is also well-understood.

We analyzed the patients with FLD who were mispredicted in the test set and found that their indicators were basically normal. We think that these people may be patients with AFLD or patients with mild FLD, they often do not have obvious symptoms and indicators change (1). Our data set does not include the alcohol intake and body condition of patients, which limits our prediction ability, because we can not exclude the interference of AFLD and we can not use the waist circumference of patients to judge whether they are obese(Even so, the AUC of our model is still high). But because of this, our model can be directly applied to the electronic physical examination records of the current health database for large-scale epidemics screening.

## 5. Conclusion

In the present study, we use the electronic physical examination records in the health database as data support, use the chi-square binning algorithm to help fill in the missing values, and use the genetic algorithm as the optimization algorithm for feature engineering, which tentatively solves the two disadvantages of the large-scale electronic medical record–missing values and lack of features. In the end, this study established an FLD prediction model based on the XGBoost algorithm with an AUC of 0.89. The satisfactory performance of the model makes large-scale screening of FLD possible, but due to the limited data breadth, more data is needed for external verification before applications.

## Data Availability Statement

The raw data supporting the conclusions of this article will be made available by the authors, without undue reservation.

## Ethics Statement

Ethical review and approval was not required for the study on human participants in accordance with the local legislation and institutional requirements. Written informed consent to participate in this study was provided by the participants' legal guardian/next of kin. Written informed consent was not obtained from the individual(s), nor the minor(s)' legal guardian/next of kin, for the publication of any potentially identifiable images or data included in this article.

## Author Contributions

MZ puts forward the idea and realizes it, completes the visualization and the writing of the paper. CS is responsible for data sorting and data analysis. TL and TH assisted in writing the first draft of the paper. SL leads research and conduct thesis writing guidance. All authors contributed to the article and approved the submitted version.

## Conflict of Interest

The authors declare that the research was conducted in the absence of any commercial or financial relationships that could be construed as a potential conflict of interest.
